# Deletion of LRP5 in VLDLR Knockout Mice Inhibits Retinal Neovascularization

**DOI:** 10.1371/journal.pone.0075186

**Published:** 2013-09-13

**Authors:** Chun-hong Xia, Eric Lu, Jing Zeng, Xiaohua Gong

**Affiliations:** School of Optometry and Vision Science Program, University of California Berkeley, Berkeley, California, United States of America; Indiana University College of Medicine, United States of America

## Abstract

The development and maintenance of retinal vasculature require a precise balance between pro-angiogenic and anti-angiogenic factors. However, mechanisms underlying normal homeostasis of retinal vasculature and pathological changes of disrupted retinal vessel development are not fully understood. Recent studies of the low-density lipoprotein receptor-related protein 5 (LRP5) and the very low-density lipoprotein receptor (VLDLR) mutant mice indicate that LRP5 mediates a pro-angiogenic signal while VLDLR mediates an anti-angiogenic signal in retinal vasculature. Mice with a loss of LRP5 display underdeveloped intraretinal vasculature associated with endothelial cell (EC) clustering and failed EC migration into deep retinal layers. In contrast, VLDLR knockout mice show overgrown intraretinal vasculature and subretinal neovascularization. To understand the mechanisms for the opposite retinal vascular abnormalities between LRP5 and VLDLR mutant mice and to test how a loss of LRP5 perturbs subretinal neovascularization caused by a loss of VLDLR, we have generated and characterized the retinal vasculature in LRP5/VLDLR double knockout (DKO) mice. Our data show that DKO mice develop substantial EC clustering without subretinal neovascularization. The absence of subretinal neovascularization in DKO mice is associated with inhibited migration of ECs into the photoreceptor cell layer. In addition, the transcription level of Slc38a5, which encodes a Müller cell specific glutamine transporter, is significantly reduced in DKO mice, similar to previously reported changes in LRP5 single knockout mice. Thus, LRP5 signaling is a prerequisite for neovascularization in VLDLR knockout mice. LRP5 may be an effective target for inhibiting intraretinal neovascularization.

## Introduction

Retinal vascular disorders remain one of the leading causes of blindness. It is necessary to explore new effective targets other than VEGF for inhibiting neovascularization caused by diabetic retinopathy, retinopathy of prematurity (ROP) and age-related macular degeneration (AMD). Wnt signaling is one of the key regulators in retinal vasculature development [1]. The low-density lipoprotein receptor-related protein 5 (LRP5), a member of the low-density lipoprotein (LDL) receptor family, is a co-receptor of the Wnt ligand-receptor complex that consists of Norrin and Frizzled 4 (FZD4) as well as an auxiliary membrane protein tetraspanin, Tspan12. Loss-of-function mutations in Norrin, FZD4, LRP5 and Tspan12 all cause familial exudative vitreoretinopathy (FEVR) in humans [2-7]. Hypovascularization of human and mouse retinas caused by mutations of these genes demonstrates the essential roles of these molecules in retinal vasculature development and homeostasis. A recent study using chimeric vasculature experiments in FZD4 conditional knockout mice reveals that this pathway is dispensable in mature retinal vasculature [8]. Thus, Norrin, FZD4, LRP5 and Tspan12 may be effective clinical targets for inhibiting neovascularization in the retina [9].

The very low-density lipoprotein receptor (VLDLR) also belongs to the LDL receptor family. It is known to mediate the binding and uptake of apoE-containing lipoproteins, such as VLDL and β-VLDL [10]. Unexpectedly, homozygous VLDLR knockout mice have normal lipoprotein profiles without any obvious phenotypes except a slightly smaller and leaner size at a young age [11]. The cause of smaller body size in young homozygous VLDLR knockout mice remains unclear. Interestingly, subretinal neovascularization has been reported to be the main phenotype in VLDLR knockout and mutant mice [12-16]. Subretinal neovascularization in VLDLR knockout mice is caused by overgrown intraretinal vasculature that leads to retinal-choroidal anastomosis, subretinal fibrosis, retinal pigment epithelium hyperplasia and photoreceptor degeneration [12-16]. A recent study indicates that the loss of VLDLR facilitates proliferation, migration and capillary-like formation of retinal vascular endothelial cells (ECs) and enhances angiogenic properties of ECs *in vitro* and *in vivo* [17]. Thus, VLDLR probably mediates negative regulation that prevents the migration of retinal ECs into the photoreceptor cell layer and subretinal space. To date, the mechanism for how VLDLR mutations lead to subretinal neovascularization is not well understood and the role of VLDLR in EC migration is unclear.

Our current study aims to elucidate the underlying mechanisms for the opposite intraretinal angiogenesis phenotypes caused by LRP5 and VLDLR mutations, to examine the roles of LRP5 and VLDLR in retinal EC migration, and to evaluate the therapeutic potential of using LRP5 as a target for preventing intraretinal neovascularization by characterizing LRP5/VLDLR double knockout (DKO) mice. We have tested whether LRP5 is crucial or dispensable for the neovascularization caused by the deletion of VLDLR. Our data reveal that neovascularization does not occur in the photoreceptor cell layer and/or the subretinal space in DKO mice. This work supports the notion that LRP5 is a prerequisite for subretinal neovascularization developed in VLDLR knockout mice, and LRP5 may be an effective target for inhibiting intraretinal neovascularization.

## Results

### The generation of *Vldlr* ^*-/-*^ *Lrp5* ^*-/-*^ double knockout (DKO) mice

**Figure 1:**
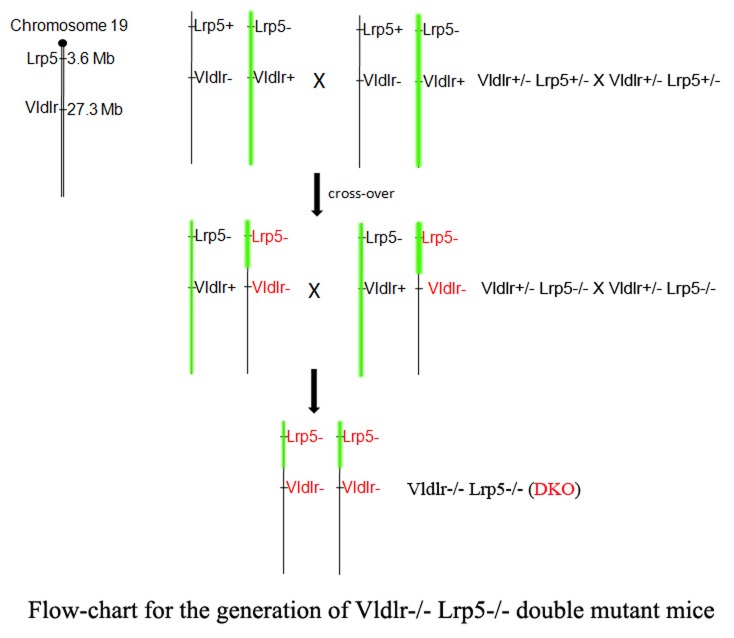
The generation of *Vldlr* ^*-/-*^ *Lrp5* ^*-/-*^ (DKO) mice via crossover mice containing both the *Lrp5* knockout allele (Lrp5^-^) and the *Vldlr* knockout allele (Vldlr^-^). The *Lrp5* and *Vldlr* genes are both located on mouse chromosome 19. Double heterozygous *Vldlr* ^*+/-*^ *Lrp5* ^*+/-*^ mice were generated by mating the *Vldlr* ^*-/-*^ and *Lrp5* ^*-/-*^ mice, and they were then intercrossed. A *Vldlr* ^*+/-*^ *Lrp5* ^*-/-*^ male mouse with a crossover was identified from 124 offspring mice. This male mouse was mated with *Lrp5* ^*-/-*^ female mice to generate both female and male *Vldlr* ^*+/-*^ *Lrp5* ^*-/-*^ mice. The DKO mice were generated by an intercross between *Vldlr* ^*+/-*^ *Lrp5* ^*-/-*^ mice.

Since the *Lrp5* and the *Vldlr* genes are located about 24 Mb apart from each other on mouse chromosome 19, we relied on a chromosomal crossover to generate the *Vldlr* ^*-/-*^ *Lrp5* ^*-/-*^ double knockout (DKO) mice. A flow-chart shows the generation of DKO mice through a crossover offspring (Figure 1). Double heterozygous mice (*Vldlr* ^*+/-*^ *Lrp5* ^*+/-*^) were generated by mating the *Vldlr* ^*-/-*^ and the *Lrp5* ^*-/-*^ single knockout mice. A male *Vldlr* ^*+/-*^ *Lrp5* ^*-/-*^ mouse founder, containing both the *Lrp5* knockout and the *Vldlr* knockout on the same chromosomal allele due to a crossover on chromosome 19, was identified from a screen of 124 offspring from the intercross of heterozygous *Vldlr* ^*+/-*^ *Lrp5* ^*+/-*^ mice. The male founder was further bred with female *Lrp5* ^*-/-*^ knockout mice to generate more *Vldlr* ^*+/-*^ *Lrp5* ^*-/-*^ mice. The *Vldlr* ^*-/-*^ *Lrp5* ^*-/-*^ double knockout (DKO) mice were produced by intercrossing *Vldlr* ^*+/-*^ *Lrp5* ^*-/-*^ mice. DKO mice are viable and fertile without other noticeable phenotypes.

### The deletion of *Lrp5* in *Vldlr* knockout mice suppresses retinal neovascularization

**Figure 2:**
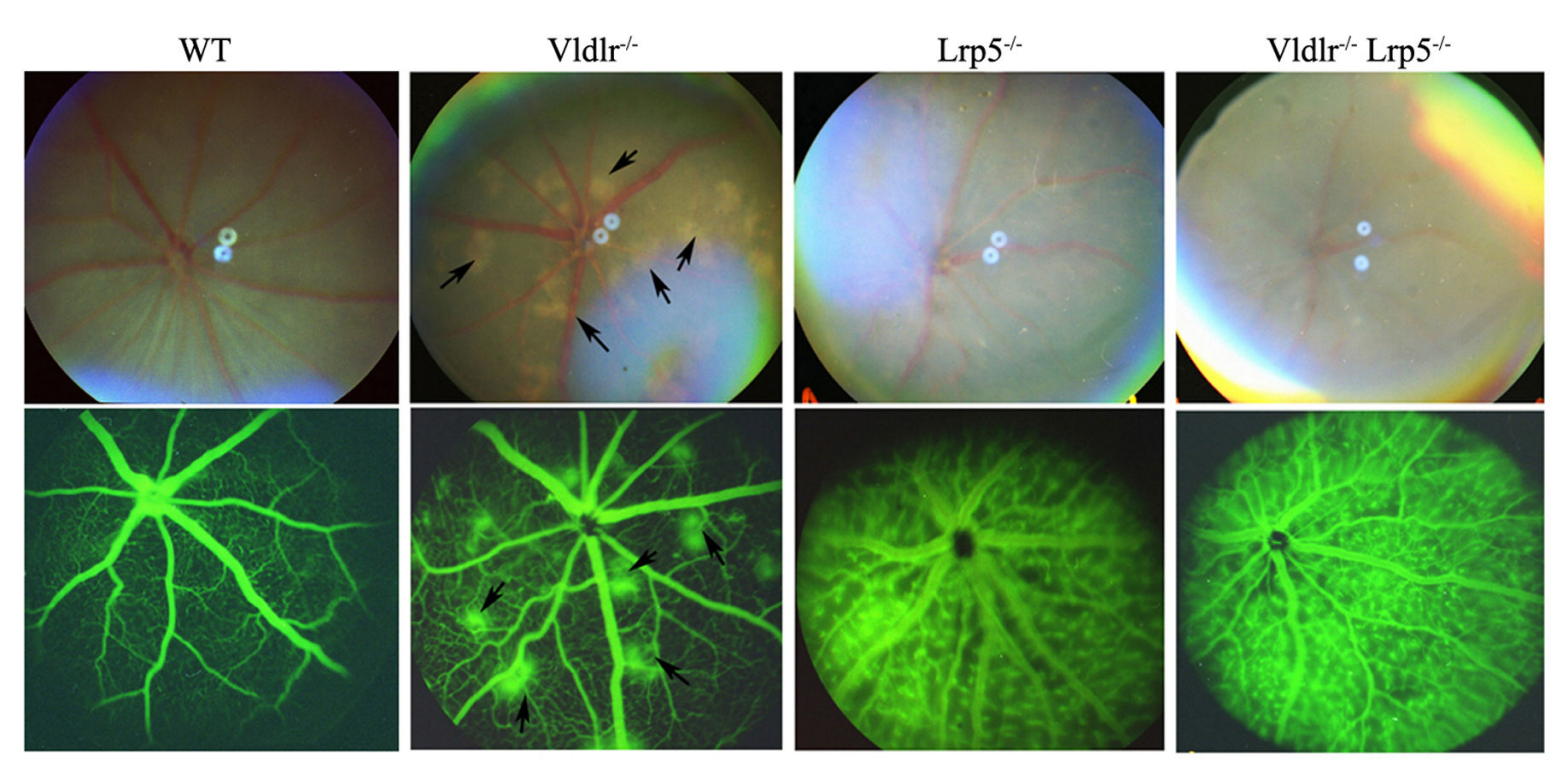
Fundus photos and fluorescein angiograms of 6-week-old wild-type (WT), *Vldlr* ^*-/-*^, *Lrp5* ^*-/-*^ and *Vldlr* ^*-/-*^ *Lrp5* ^*-/-*^ or DKO mice. The upper panels are fundus photos, and the lower panels are angiograms. Hypopigmented yellowish patches in the fundus of *Vldlr* ^*-/-*^ (arrows) were not observed in *Lrp5* ^*-/-*^ or DKO fundus. The *Vldlr* ^*-/-*^ angiogram displayed hyperfluorescent spots (arrows) that corresponded to hypopigmented yellowish patches on the fundus. Both *Lrp5* ^*-/-*^ and DKO angiograms showed homogeneous leakage of retinal vasculature without hyperfluorescent spots.

Fundus examination and fluorescein angiography were used to evaluate the clinical changes of retinal vasculature in DKO mice. We compared fundus photos and fluorescein angiogram pictures of DKO mice with age-matched *Vldlr* ^*-/-*^ and *Lrp5* ^*-/-*^ single knockout mice (Figure 2). Retinal phenotypes of both single knockout mice were identical to previous reports [12-15,18]. *Vldlr* ^*-/-*^ mice displayed hypopigmented yellowish patches on the fundus, which match the hyperfluorescent spots on the angiogram; *Lrp5* ^*-/-*^ mice showed attenuated retinal arteries on the fundus and homogenous leakage of retinal vessels on the angiogram (Figure 2). The fundus photo of DKO mice showed attenuated retinal arteries without hypopigmented yellowish patches, and the fluorescein angiogram of DKO mice revealed homogenous leakage of retinal vessels without hyperfluorescent spots (Figure 2). Thus, DKO retinal phenotypes resembled that of *Lrp5* ^*-/-*^ mice but obviously differed from that of *Vldlr* ^*-/-*^ mice. These data suggest that a lack of LRP5 probably suppresses subretinal neovascularization caused by a loss of VLDLR in DKO mice.

**Figure 3:**
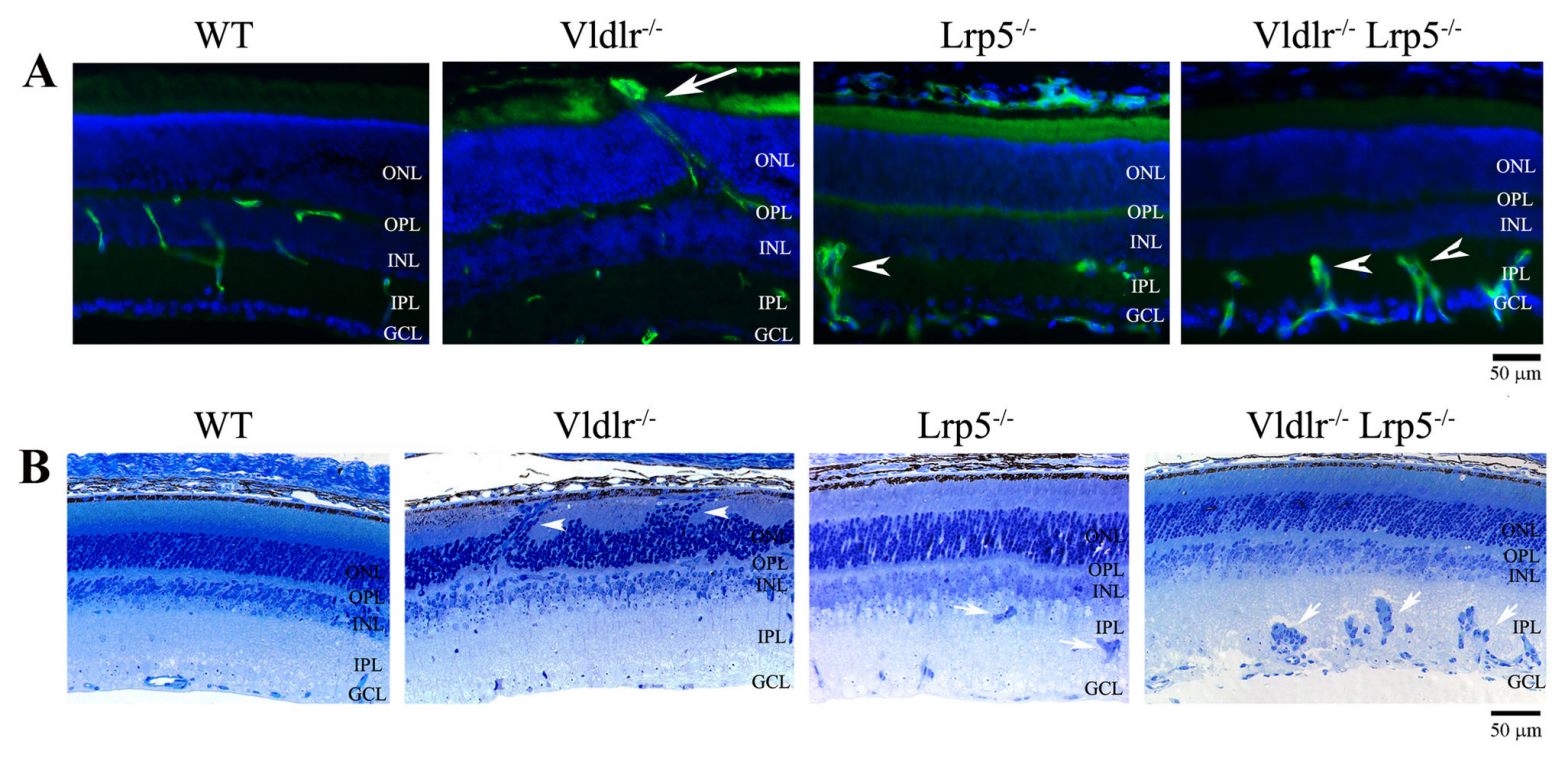
The deletion of LRP5 prevents the overgrowth of retinal vessels in the *Vldlr* ^*-/-*^ mice. (**A**) Immunostaining of 3-week-old WT, *Vldlr* ^*-/-*^, *Lrp5* ^*-/-*^ and *Vldlr* ^*-/-*^ *Lrp5* ^*-/-*^ DKO retinal sections. Retinal vessels were visualized with an anti-CD31 antibody (green), and cell nuclei were labeled by DAPI (blue). In the WT control retina, vascular ECs were observed in the outer-plexiform layer (OPL), inner-plexiform layer (IPL), and ganglion cell layer (GCL). In the *Vldlr* ^*-/-*^ retina, CD31 signals were not only present in the OPL, IPL and GCL, but also in the photoreceptor outer nuclear layer (ONL) and in the subretinal space (white arrow). In the *Lrp5* ^*-/-*^ retina, very few CD31-stained cells were detected in the OPL, and CD31-positive ECs formed clusters in the IPL (white arrowhead). A CD31 staining pattern similar to *Lrp5* ^*-/-*^ was observed in the DKO retina, where ECs also formed clusters (white arrowheads). INL: inner nuclear layer. Scale bar: 50µm. (**B**) Retinal histology of 3-week-old WT, *Vldlr* ^*-/-*^, *Lrp5* ^*-/-*^, and DKO mice. Compared to the WT retinal section, the *Vldlr* ^*-/-*^ section displayed a disorganized ONL with photoreceptor cells moving along the abnormal vessels (white arrowheads). Both *Lrp5* ^*-/-*^ and DKO sections showed toluidine blue-stained cell clusters (white arrows) in the IPL. Scale bar: 50µm.

We further characterized the retinal vasculature of DKO mice by preforming histological analysis and immunostaining of frozen retinal sections with specific vascular markers. Immunostaining of CD31, a vascular EC-specific marker, revealed that retinal vessels were localized in the ganglion cell layer (GCL), inner-plexiform layer (IPL) and outer-plexiform layer (OPL), but never in the photoreceptor cells and retinal pigment epithelium (RPE) in wild-type mice (Figure 3A). However, the *Vldlr* ^*-/-*^ retinal section showed that CD31-positive vascular ECs not only appeared in normal locations of GCL, IPL and OPL, but also in the photoreceptor cell layer (ONL) and subretinal space next to the RPE (Figure 3A). In contrast, the *Lrp5* ^*-/-*^ mutant retina showed underdeveloped vasculature with clustered ECs in the IPL and very few CD31-positive ECs in the OPL (Figure 3A), as we reported previously [18,19]. The DKO retina had many CD31-positive EC clusters in the IPL, very few CD31-positive ECs in the OPL and no CD31-positive ECs in either the ONL or subretinal space. Thus, CD31 staining results support the notion that vasculature in the DKO retina is similar to that of the *Lrp5* ^*-/-*^ retina, but differed from that in the *Vldlr* ^*-/-*^ retina.

Histology analysis was performed to compare the retinal morphology of wild-type, *Vldlr* ^*-/-*^, *Lrp5* ^*-/-*^ and DKO mice (Figure 3B). The *Vldlr* ^*-/-*^ retina displayed a distorted ONL (Figure 3B, white arrowheads), likely resulting from abnormal vascular overgrowth. The *Lrp5* ^*-/-*^ retina showed a normal ONL and dark toluidine blue-stained cell clusters in the IPL (Figure 3B, white arrows). The DKO retina displayed substantial toluidine blue-stained cell clusters in the IPL and a normal photoreceptor cell layer without noticeable change in the ONL (Figure 3B). Presumably, the dark toluidine blue-stained cell clusters in IPL of the *Lrp5* ^*-/-*^ and DKO retinas were the same as the CD31-positive EC clusters (Figure 3A). Retinal histopathology of DKO mice was similar to that of *Lrp5* ^*-/-*^ mice. Moreover, DKO retinas lacked subretinal neovascularization caused by the loss of VLDLR. Therefore, we hypothesized that abnormal cell clusters in DKO retinas resulted from an inhibition of EC migration into the deep retinal layers caused by the loss of LRP5. We further tested this hypothesis by characterizing the development of retinal angiogenesis using GFP-positive ECs *in vivo*.

### Retinal angiogenesis monitored by GFP-positive ECs

**Figure 4:**
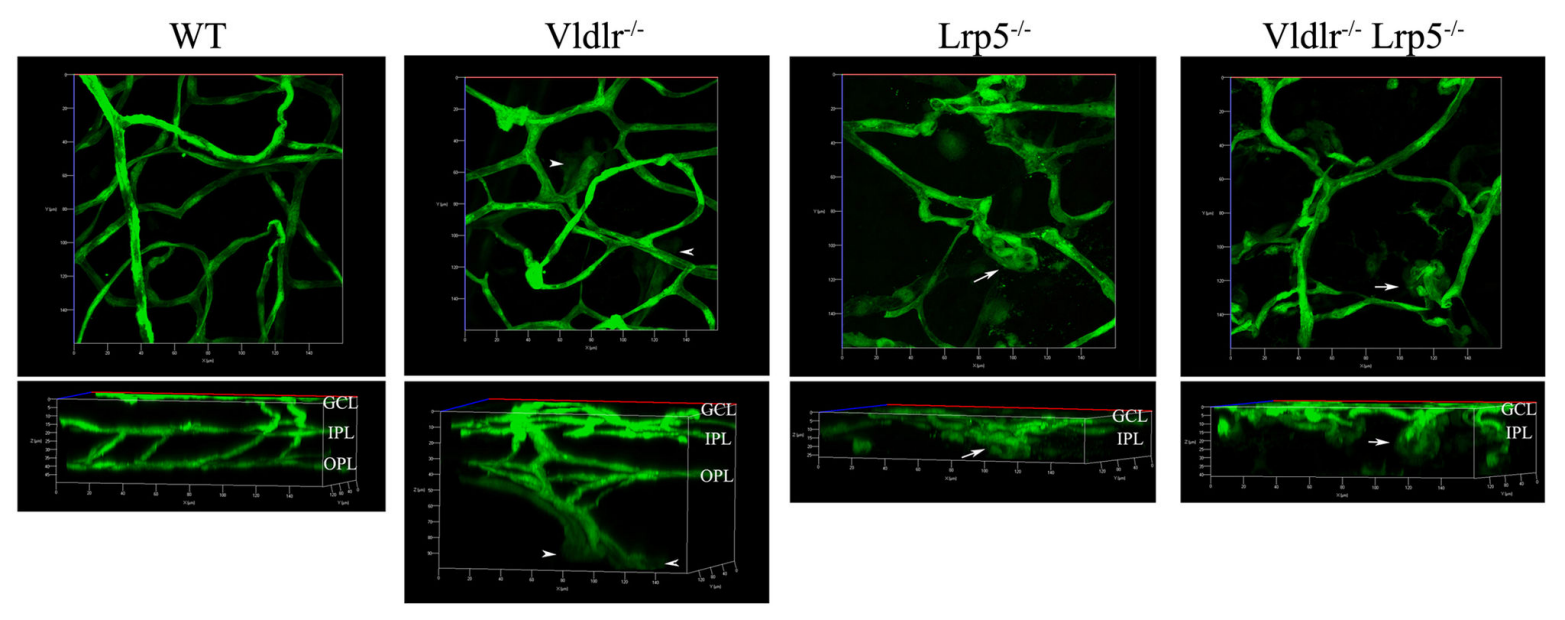
3D reconstruction of retinal vasculature in 3-week-old WT, *Vldlr* ^*-/-*^, *Lrp5* ^*-/-*^ and DKO mice with the Sca1-GFP transgene. The 3D images were constructed from Z-stack images of GFP-positive ECs, starting from the retinal surface (GCL) through the OPL. The upper panels show a top view of 3D images while the lower panels show a 90 degree–rotated side view of the upper 3D images. The top-view panel (the upper left) reveals wild-type retinal vasculature consisting of large vessels, small vessels and capillaries while the side-view panel (the lower left) shows a typical interconnected 3-layer of retinal vasculature, corresponding to enriched vessels in the GCL (upper), IPL (middle) and OPL (lower), in the wild-type retina. Compared to a clear 3-layer retinal vascular network in the WT control, the *Vldlr* ^*-/-*^ retina shows abnormal vessel growth originated from the OPL vessels (white arrowheads). The *Lrp5* ^*-/-*^ and DKO retinas display very similar phenotypes, such as incomplete retinal vasculature with only the surface vessel network and the formation of EC clusters in the IPL (white arrows).

Our recent work showed that retinal angiogenesis during development could be monitored with the restrictive expression of GFP in retinal ECs from the Sca1-GFP transgene [19-22]. Confocal images of GFP-positive EC distribution in the retina provide a way to monitor EC migration and network formation during retinal angiogenesis in mice [19]. As expected from the results of CD31 immunostaining and histology, GFP-positive ECs clustered in the IPL (Figure 4, white arrows) and failed to spread out and migrate into deeper layers to form vascular networks in the *Lrp5* ^*-/-*^ retina. In contrast, *Vldlr* ^*-/-*^ GFP-positive ECs migrated into ONL and the subretinal space, recapitulating vascular overgrowth and neovascularization (Figure 4, white arrowheads). In the DKO retina, GFP-positive ECs clustered extensively in the IPL (Figure 4, white arrows) and failed to form appropriate vascular networks in either the IPL or the OPL. There were no GFP-positive ECs in the photoreceptor cell layer or the subretinal space of the DKO retina. Thus, the clustering of ECs caused by the migration defect due to a functional loss of LRP5 is responsible for the prevention of subretinal neovascularization caused by a functional loss of VLDLR in DKO mice. We further investigated the expression and distribution of some angiogenesis-related molecules as well as the downstream targets of LRP5 and VLDLR signaling that may regulate EC migration needed for the development of retinal vasculature.

### Transcription levels of angiogenesis-related molecules

We examined whether key angiogenesis-related molecules were altered in *Vldlr* ^*-/-*^, *Lrp5* ^*-/-*^ and DKO mutant retinas by using semi-quantitative PCR and quantitative real-time PCR (RT-qPCR) analyses. Retinal RNAs from each individual mouse were reverse-transcribed to make cDNAs, and specific primer pairs were designed and used to amplify PCR products for each molecule examined.

**Figure 5:**
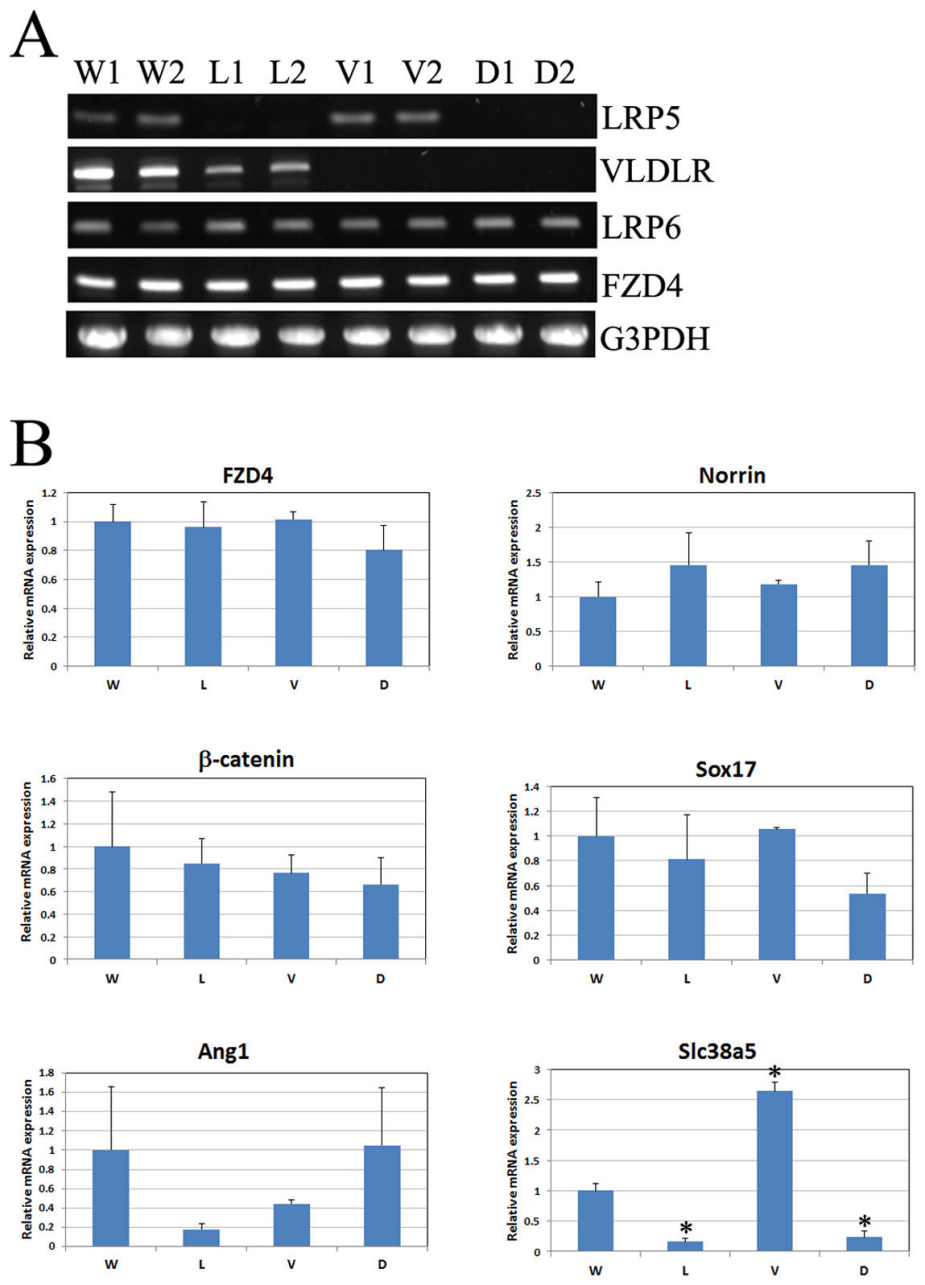
Transcription levels of angiogenesis-related molecules examined by semi-quantitative PCR and quantitative real-time PCR. (**A**) Representative semi-quantitative RT-PCR results from 3-week-old WT, *Lrp5* ^*-/-*^, *Vldlr* ^*-/-*^ and DKO mice. Results from retinal RNAs of two littermate mice for each genotype were shown. As expected, *Lrp5* ^*-/-*^ retinas (L1 and L2) lacked detectable LRP5 transcript and *Vldlr* ^*-/-*^ retinas (V1 and V2) showed no expression of VLDLR. Both LRP5 and VLDLR transcripts were undetectable in DKO retinas (D1 and D2). Moreover, transcription levels of both Lrp6 and FZD4 remained unchanged among WT, *Lrp5* ^*-/-*^, *Vldlr* ^*-/-*^ and DKO retinas. The G3PDH levels from the same RNA samples were used as quantification controls. (**B**) Relative expression levels of FZD4, Norrin, β-catenin, Sox17, Ang1 and Slc38a5 in retinas of 3-week-old WT (W), *Lrp5* ^*-/-*^ (L), *Vldlr* ^*-/-*^ (V) and DKO (D) mice were examined by RT-qPCR. The bar graphs show relative changes of these molecules compared to WT. Average WT levels normalized to G3PDH were set as 1. Retinas from three littermate mice of same genotype (n=3) were used for the study. *P<0.05.

Representative semi-quantitative PCR data from 3-week-old wild-type, *Lrp5* ^*-/-*^, *Vldlr* ^*-/-*^ and *DKO* mice showed that the transcription levels of Wnt-receptor FZD4 and co-receptor LRP6 remained unchanged between wild-type and mutant samples (Figure 5A), while the transcription level of β-catenin, a downstream target of Wnt signaling pathway, was slightly decreased in *Lrp5* ^*-/-*^, *Vldlr* ^*-/-*^ and *DKO* retinas (data not shown). Expression levels of VEGF, VEGFR1, VEGFR2, Notch1, delta-like 4 (Dll4), thrombospondin-1 (TSP1), ephrin-B2 and EphB4 showed no significant changes between wild-type and different mutant retinal samples (data not shown).

The RT-qPCR data confirmed our semi-quantitative PCR results of FZD4, and further revealed a slight reduction of β-catenin expression in *Lrp5* ^*-/-*^ and DKO mutant retinas but the changes were not statistically significant (p>0.05) (Figure 5B). The Wnt ligand Norrin seemed to be increased in *Lrp5* ^*-/-*^ and DKO retinas although the changes were not statistically significant (p>0.05). Sox17 expression showed a slight decrease in both *Lrp5* ^*-/-*^ and DKO retinas, but was unchanged in *Vldlr* ^*-/-*^ retinas; angiopoietin-1 (Ang1) expression was reduced in *Lrp5* ^*-/-*^ and *Vldlr* ^*-/-*^ retinas but unchanged in *DKO* samples; however, changes of Sox17 and Ang1 expression in different mutant retinas were not statistically significant (p>0.05) when compared to wild-type controls. Significant changes were only found for Slc38a5 gene (solute carrier family 38, member 5), encoding a Müller cell specific glutamine transporter. Previous studies have shown significantly reduced Slc38a5 expression in LRP5 knockout retinas [19,23]. Our current data revealed significantly reduced Slc38a5 expression in both *Lrp5* ^*-/-*^ and DKO retinas; interestingly, Slc38a5 expression was significantly increased in *Vldlr* ^*-/-*^ retinas. Thus, the downregulation and upregulation of Slc38a5 expression correlated with distinct changes of retinal angiogenesis among *Vldlr* ^*-/-*^, *Lrp5* ^*-/-*^ and DKO mice. Since retinal vascular leakage is another profound phenotype observed in all three mutant mice, we have further investigated a specific fenestrated EC marker, the plasmalemmal vesicle-associated protein (PLVAP, or MECA-32), known to be associated with vascular leakage in mutant retinas [24].

### Increased retinal vessel permeability in DKO retinas

**Figure 6:**
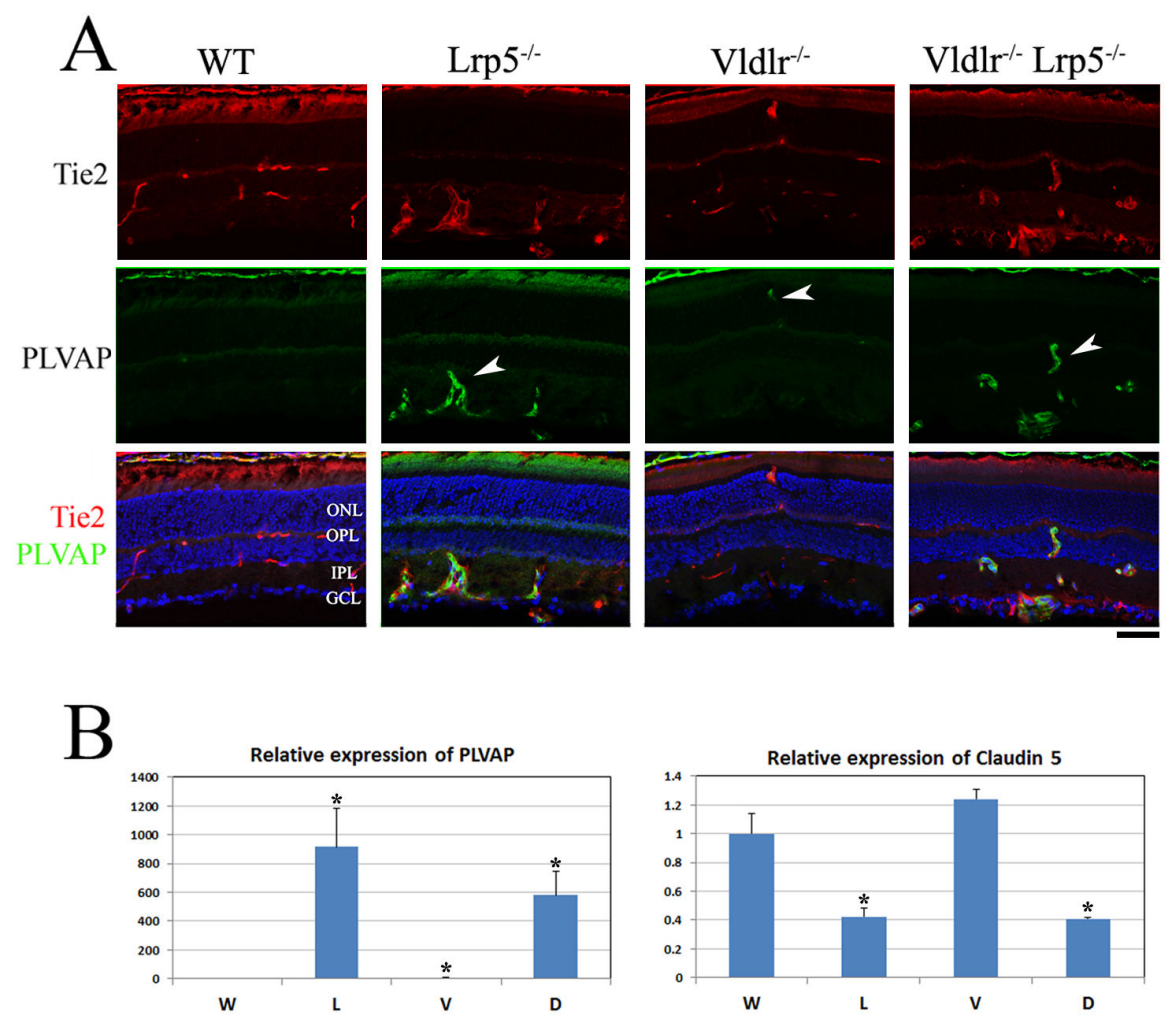
Expression of PLVAP and claudin-5 in WT, *Lrp5* ^*-/-*^, *Vldlr* ^*-/-*^ and DKO retinas. (**A**) Immunostaining of 3-week-old WT, *Lrp5* ^*-/-*^, *Vldlr* ^*-/-*^ and DKO retinal frozen sections. An antibody against PLVAP (green) was co-stained with a vascular EC-specific Tie2 antibody (red). In the WT section, PLVAP expression was undetectable in retinal ECs. PLVAP staining signals was obviously increased in retinal ECs of both *Lrp5* ^*-/-*^ and DKO mice (arrowheads). However, the PLVAP staining signals was restrictively detectable only in abnormal overgrown vessels located in the ONL in the *Vldlr* ^*-/-*^ retina (arrowhead). Scale bar: 50µm. (**B**) Relative expression of PLVAP and claudin-5 transcripts in WT (W), *Lrp5* ^*-/-*^ (L), *Vldlr* ^*-/-*^ (V) and DKO (D) retinas examined by qRT-PCR. PLVAP expression was significantly increased in both *Lrp5* ^*-/-*^ and DKO retinas, as well as in *Vldlr* ^*-/-*^ retinas. Significantly reduced claudin-5 expression was observed in both *Lrp5* ^*-/-*^ and DKO retinas, while the claudin-5 expression in *Vldlr* ^*-/-*^ retinas seemed to be slightly increased although this change is not statistically significant. *p<0.05, n=3 for each genotype.

Normal retinal ECs have no fenestration and utilize intercellular tight junctions to form the blood-retinal barrier; thus PLVAP, a fenestrated EC marker, has very low or undetectable expression in the normal retinal vasculature. We examined the localization and expression of the PLVAP protein in retinal frozen sections of 3-week-old wild-type*, Lrp5* ^*-/-*^, *Vldlr* ^*-/-*^ and DKO mice by co-staining with specific antibodies against PLVAP and Tie2, a vascular EC-specific marker. In the wild-type retinal section, Tie2 signals were detected in retinal vessels, while PLVAP was undetectable in retinal ECs (Figure 6A). However, strong signals of PLVAP protein staining were detected in ECs of both *Lrp5* ^*-/-*^ and DKO retinas (Figure 6A, white arrows). In the *Vldlr* ^*-/-*^ retina, noticeable PLVAP staining signals were detected in ECs that were particularly associated with the neovascularization in OPL and subretinal spaces (Figure 6A, white arrow). The transcription expression of PLVAP was further examined. The RT-qPCR data showed that PLVAP expression was barely detectable in wild-type retinal samples but was significantly increased in *Lrp5* ^*-/-*^ and DKO retinas, ~900-fold and ~600-fold, respectively (Figure 6B). The *Vldlr* ^*-/-*^ retinas also showed some upregulation of PLVAP, ~5-fold (p<0.05). Thus, drastic upregulation of PLVAP in retinal ECs was associated with profound vascular leakage in *Lrp5* ^*-/-*^ and DKO retinas; restrictive upregulation of PLVAP probably correlated to the leaky spots of subretinal neovascularization in the *Vldlr* ^*-/-*^ retina.

We also examined the expression of claudin-5, one of the key components of tight junctions in retinal ECs, by RT-qPCR (Figure 6B). *Lrp5* ^*-/-*^ and DKO retinas displayed significantly reduced expression of claudin-5(p<0.05), while claudin-5 expression seemed to be increased in *Vldlr* ^*-/-*^ retinas although the change is not statistically significant. The downregulation of claudin-5 reflects an impairment of tight junction formation between retinal ECs, which may directly contribute to profound leakage of *Lrp5* ^*-/-*^ and DKO retinal vasculature.

## Discussion

**Figure 7:**
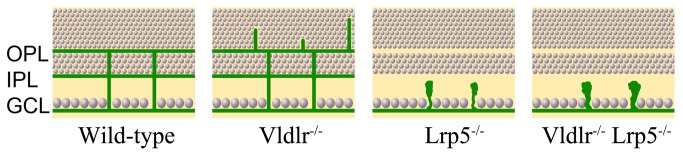
A schematic summary of retinal vasculature phenotypes in *Vldlr* ^*-/-*^, *Lrp5* ^*-/-*^, and *Vldlr* ^*-/-*^ *Lrp5* ^*-/-*^ DKO mice. Compared to the typical three-layer retinal vasculature network (green) in GCL, IPL and OPL of the wild-type control retina, the *Vldlr* ^*-/-*^ retina displays abnormal vessel growth into the photoreceptor nuclear layer, which originated from retinal vessels in the OPL layer; the *Lrp5* ^*-/-*^ retina has the surface vessel layer in the GCL but has no vascular networks extending to the IPL and OPL, and ECs form cell clusters in IPL. Similar to the *Lrp5* ^*-/-*^ retina, the DKO retina does not form a complete retinal vascular network except the surface layer in the GCL, and the DKO retina also forms large ECs clusters in IPL.

Previous studies have demonstrated that a loss of LRP5 causes underdeveloped retinal vasculature, whereas a lack of VLDLR leads to retinal neovascularization [12-15,18]. This work reveals that *Lrp5* ^*-/-*^ *Vldlr* ^*-/-*^ DKO mice show no subretinal neovascularization, which occurred in *Vldlr* ^*-/-*^ mice; the DKO mice develop incomplete retinal vasculature, resembling the phenotype of *Lrp5* ^*-/-*^ mice (Figure 7). This key finding suggests that the pro-angiogenic signal mediated by LRP5 is a prerequisite for the anti-angiogenic signal mediated by VLDLR in the retina. The inhibition of subretinal neovascularization in DKO mice is associated with the prevention of retinal EC migration into the photoreceptor cell layer and subretinal space. Thus, LRP5 may be an effective and specific target for preventing or treating retinal diseases caused by neovascularization in the photoreceptor cell layer and subretinal space.

The mechanisms underlying the regulation and patterning of the intraretinal vasculature are not fully understood. The retinal vasculature has a stereotyped architecture composed of one surface vessel plexus and two intraretinal capillary networks in IPL and OPL. The primary vessel plexus is formed by ECs sprouting from the optic nerve head and spreading to the periphery on the surface of the GCL. In mice, a gradient of VEGF, laid down by astrocytes, stimulates the migration of capillary tip cells and the proliferation of trailing stalk cells to induce primary vasculature along the pre-existing astrocyte network in the first postnatal week [25,26]. The two intraretinal capillary networks are subsequently generated as new tip cells emerge and vertically sprout into the deeper layers of retina [27,28]. A critical step of retinal angiogenesis is the selection of a tip cell to lead the sprout into deeper retinal layers to complete a patterned vasculature at about three weeks of age in mice. Many signaling molecules, such as VEGF/VEGFR, Delta/Notch and ephrin-B2/EphB4, have been studied for their roles in the regulation of tip cells and/or stalk cells of retinal surface primary vasculature [29-35]. However, the cellular and molecular mechanisms that regulate the formation of intraretinal vascular networks in the OPL and IPL remain poorly understood. The opposite intraretinal vascular defects make the LRP5 and VLDLR knockout mice valuable models for studying intraretinal angiogenesis, and the generation of DKO mice allows us to investigate the interaction between these two signaling pathways.

LRP5 single knockout and DKO mice develop a relatively normal retinal surface vessel plexus. However, both LRP5 knockout [19] and DKO mice are unable to form vascular capillary networks in deeper retinal plexiform layers (OPL and IPL). Our previous study has suggested that LRP5 signaling is essential for regulating the sprouting, migrating and/or anastomosing of ECs into deeper layers of the retina [19]. This work further demonstrates that the clustering of vascular ECs is also the cause of incomplete retinal vasculature in the deeper layers of DKO mutant retina. DKO mice develop abnormal retinal vasculature resembling that of LRP5 knockout mice. Thus, LRP5 signaling is more dominant than the VLDLR signaling in the regulation of EC sprouting and migration in the retina.

The LRP5 protein is a co-receptor in the canonical Wnt-signaling cascade, its absence in the knockout mice would presumably affect the downstream activation of Wnt signaling, such as β-catenin activation. Due to the ubiquitous expression of β-catenin and a lack of an antibody that detects only active form of β-catenin, we were unable to evaluate whether β-catenin activation is present in retinal ECs of these mutant retinas. We detected somewhat reduced β-catenin transcription levels in *Lrp5* ^*-/-*^ and DKO retinas, and this may lead to the downregulation of β-catenin signaling events during retinal angiogenesis. Sox17, a downstream mediator of angiogenesis by Norrin/FZD4/LRP5 signaling and significantly suppressed in *Fzd4* ^*-/-*^ mice [8], is slightly downregulated in *Lrp5* ^*-/-*^ and DKO retinas. It is possible that β-catenin signaling and Sox17 may play a role in the inhibition of retinal angiogenesis.

DKO retinas show prominent reduction of the sodium coupled neutral amino acid transporter Slc38a5 expression, similar to LRP5 mutant retinas as previously reported [19,23]. Decreased expression of Slc38a5 mRNA is also found in Norrin knockout mice [36]. These data suggest that Slc38a5 seems to be a common downstream target of Norrin/FZD4/LRP5 signaling pathway. However, Slc38a5 is expressed in retinal Müller cells, and it is mainly responsible for the glutamine uptake in retinal Müller cells [37]. Although Norrin and LRP5 are predominantly expressed in retinal Müller cells [8,19], FZD4 is restrictively utilized in retinal ECs. It seems unlikely that LRP5 in Müller cells will interact with FZD4 in endothelial cells based on current knowledge in the literature. Interestingly, *Vldlr* ^*-/-*^ retinas with overgrown vasculature display increased Slc38a5 expression. To date, VLDLR has not been suggested to be involved in the Norrin (or Wnt)/FZD signaling pathway. The downstream pathway of VLDLR signaling remains unclear. Further studies will be important to elucidate the role of Slc38a5 in intricate downstream interactions among VLDLR, LRP5 and FZD4 signaling pathways and the novel function of Müller cells in the regulation of retinal vasculature.

Plasmalemmal vesicle-associated protein (PLVAP) is a key component of fenestration in ECs that aid in selective transport of molecules [24]. PLVAP expression is upregulated in tumor endothelium by VEGF [38]. Since vessel permeability is associated with the numbers of fenestrae present in capillaries, upregulated PLVAP expression is correlated with increased retinal vasculature leakage in the LRP5 knockout and DKO mice as well as in the leaky spots of the neovascular sites in VLDLR knockout mice. Retinal vascular leakage is also correlated with the downregulation of claudin-5, one of the components of tight junctions, in LRP5 knockout [39] and DKO retinas. PLVAP upregulation and claudin-5 downregulation may synergistically promote vascular leakage in retina.

In summary, studies of DKO mice indicate that the presence of LRP5 is a prerequisite for retinal neovascularization caused by a functional loss of VLDLR. LRP5 may be an effective target to prevent intraretinal neovascularization. Combined inhibition of PLVAP upregulation and claudin-5 downregulation is probably an innovative way to prevent or treat retinal vascular leakage.

## Materials and Methods

### Mice

Animals were cared for in accordance with the ARVO statement for the Use of Animals in Ophthalmic and Vision Research, and all studies and examinations were conducted in accordance with a protocol (MAUP#: R280-1213BC) approved by the Animal Care and Use Committee (ACUC) at University of California, Berkeley. The LRP5 knockout mice [40] and VLDLR knockout mice [12] were mated to generate the DKO mice. For LRP5 knockout genotyping, a primer pair of forward 5’-GGC TGA GGA AGT GCT GCT and reverse 5’-CCC ATC ACA GGG TGC AAC produces a wild-type band (~390 bp), the insertion of the IRES-*β-galactosidase*/neo^r^ cassette before the reverse primer in the knockout mice results in an absence of the wild-type band; the common forward primer 5’-GGC TGA GGA AGT GCT GCT and a LacZ specific reverse primer 5’-CAG GGT TTT CCC AGT CAC GAC will generate a knockout band (~850 bp). VLDLR knockout mice were genotyped based on a PCR protocol from The Jackson Laboratory; the primer pair of 5’-TGG TGA TGA GAG GCT TGT ATG TTG TC and 5’-TTG ACC TCA TCG CTG CCG TCC TTG generates a wild-type band (~400 bp), and the primer pair of 5’-CGG CGA GGA TCT CGT CGT GAC CCA and 5’-GCG ATA CCG TAA AGC ACG AGG AAG results in a knockout band (~200 bp).

### Fundus Photography and Fluorescein Angiography

Fundus photography and fluorescent angiography were performed as described previously [18]. For fundus examination, mouse pupils were dilated with a mixed ophthalmic solution containing 0.5% Atropine Sulfate (E. Fougera & Co) and 1.25% Phenylephrine Hydrochloride (Wilson Ophthamic), and mouse fundus was photographed with a Kowa Genesis fundus camera for small animals (Tokyo, Japan). For fluorescein angiography, mouse pupils were dilated and mice were intraperitoneally injected with 25% Angiofluor^TM^ (Alliance Pharmaceutical, Inc.) at a dose of 0.01 ml per 5 gram of mouse body weight; photos were taken with a camera containing a barrier filter for fluorescein angiography. Kodak 800 ASA color film was used for photograph. Developed negative film was scanned and processed by Photoshop for digital images.

### Immunofluorescent Staining and Histology Analysis

Immunofluorescent staining and retinal histology analysis were performed as described previously [18]. Mouse eyes for immunostaining were fixed with 4% paraformaldehyde in PBS, while eyes for histology were fixed with a solution containing 2.5% gluteraldehyde, 2% paraformaldehyde in 0.1 M cacodylate buffer (pH 7.2). Antibodies used for immunostaining: a rat anti-CD31 antibody (BD Pharmingen), a mouse anti-Tie2 antibody (Calbiochem), and a rat anti-Meca-32 (PLVAP) antibody (BD Pharmingen). Fluorescent images were collected using a Zeiss Axiovert 200 fluorescent microscope (Figure 3) and a Zeiss LSM700 confocal microscope (Figure 6), and histology images were collected with a Zeiss Axiovert 200 light microscope with a digital camera.

### Imaging and analyzing retinal vasculature of Sca1-GFP mice

The Sca1-GFP transgene was crossed into single or double knockout mice by mating with Sca1-GFP transgenic mice [21]. Retinal vasculature was evaluated based on the three-dimensional distribution of GFP-positive ECs in whole-mount retinas of Sca1-GFP positive wild-type, *Vldlr* ^*-/-*^, *Lrp5* ^*-/-*^ and DKO mice. Briefly, mouse eyes were fixed in 4% formaldehyde/PBS, cornea and lens were removed, and the remaining eye cups were flat-mounted; Z-stack images of GFP-positive ECs were recorded with a Zeiss LSM700 confocal microscope. ZEN 2010 software was used to create three-dimensional reconstructions.

### RNA isolation and PCR analysis

Semi-quantitative PCR analysis to examine the transcription levels of various molecules was performed as described previously [19]. Two retinas from each mouse of various genotypes were dissected out and combined into one tube. RNAs were isolated using the Trizol^®^ Reagent (Invitrogen Life Technologies), and cDNAs were synthesized with the Superscript^TM^ First-Strand Synthesis System for RT-PCR kit (Invitrogen) from equal amount of total RNA for each sample. PCR was performed with the same amount of cDNA from each mouse in a 20µl of volume. PCR conditions for all primer sets were: denaturation at 94°C for 2 minutes, 30 cycles of denaturation at 94°C for 30 seconds and annealing and elongation at 68°C for 1 minute, and a final 10 minutes elongation at 72°C. Previously reported primers were used for LRP5, LRP6, FZD4, and G3PDH [19]. For VLDLR, a pair of primers 5’-AAC TTG TTG TGC GGA CGA AC and 5’-TGC ACT TGA ACT TTC CAG GG(~1200 bp) were used for PCR amplification. Glyceraldehydes-3-phosphate dehydrogenase (G3PDH) was used as a house keeping gene control. Equal volume of PCR product from each individual sample was loaded into 1% agarose gel for electrophoresis analysis.

For quantitative real-time PCR (RT-qPCR) analysis, previously reported primers were used for β-catenin [19], the primer set of forward 5’-CGC TGG CAG TAC AAT GAC AG and reverse 5’-GGT TCC TAT CTC AAG CAT GG was used for Angiopoietin-1 (Ang1) and the primer set of forward 5’-GGT ACT ACC TGC GCT ATT TC and reverse 5’-CTT CTC GCT CAG GAT GAT AG was used for Plasmalemmal vesicle-associated protein (PLVAP). Using Integrated DNA Technologies (IDT) PrimerQuest, we designed the following primers for RT-qPCR. FZD4: forward 5’-GGC TAC AAC GTG ACC AAG ATG and reverse 5’-GGC ACA TAA ACC GAA CAA AGG; Norrin: forward 5’-GTC GAT TCT ATC AGT CAC CCA C and reverse 5’-AGG TTG CTT GAG GAC AGT G; Sox17: forward 5’-CGA TGA ACG CCT TTA TGG TG and reverse 5’-TTC TCT GCC AAG GTC AAC G; Slc38a5: forward 5’-ATG GCT TTT GCG TTT GTC TG and reverse 5’-GCG GTA TCC AAA GGT CGC TG; Claudin 5: forward 5’-AAT CAA TTC CCA GCT CCC AG and reverse 5’-CAT CCT ACC AGA CAC AGC AC. The iScript^TM^ reverse transcription supermix (BIO-RAD) was used to make cDNA for RT-qPCR, and quantitative gene expression was determined using SsoAdvanced^TM^ SYBR Green Supermix (BIO-RAD) and CFX96^TM^ Real-Time System (BIO-RAD). For statistical analysis of RT-qPCR results, data were presented as mean±SEM. For each target gene, cDNAs from three individual mice (n=3) were examined for each genotype, and triplicate wells for each individual cDNA were repeated on a PCR plate. Each target gene Starting Quantity (SQ) was normalized to the amount of G3PDH and a two-sample Student’s t-Test was used to calculate the p value.
